# The role of community health workers in cervical cancer screening in low-income and middle-income countries: a systematic scoping review of the literature

**DOI:** 10.1136/bmjgh-2019-001452

**Published:** 2019-05-13

**Authors:** James O’Donovan, Charles O’Donovan, Shobhana Nagraj

**Affiliations:** 1 Department of Education, University of Oxford, Oxford, UK; 2 Health Education North West London, North West Thames Foundation School, London, UK; 3 Nuffield Department of Women's & Reproductive Health, University of Oxford, Oxford, UK; 4 The George Institute for Global Health, Oxford, UK

**Keywords:** cancer, screening, public health, systematic review, maternal health

## Abstract

**Introduction:**

Community-based screening for cervical cancer and task sharing to community health workers (CHWs) have been suggested as a potential way to increase screening coverage in low- and middle-income countries (LMICs). The aims of the scoping review were to understand the following: (i) where and how CHWs are currently deployed in screening in LMIC settings; (ii) the methods used to train and support CHWs in screening, and (iii) The evidence on the cost-effectiveness of using CHWs to assist in screening.

**Methods:**

A scoping literature search of 11 major databases and the grey literature was performed between 1978 and 2018. We included comprehensive search terms for ‘CHWs’ and ‘Cervical Cancer’, and used the World Bank criteria to define LMICs.

**Results:**

Of the 420 articles screened, 15 met the inclusion criteria for review. Studies were located in Africa (n=5), Asia (n=5), and South and Central America (n=5). CHWs played a role in community education and raising awareness (n=14), conducting or assisting in cervical screening (n=5), or follow-up (n=1). 11 studies described CHW training activities. Only one study provided a formal cost analysis.

**Conclusion:**

The roles of CHWs in cervical cancer screening in LMICs have largely to date focused on education, outreach, and awareness programmes. Community-based approaches to cervical cancer screening are feasible, although the sociocultural context plays an important role in the acceptability of these interventions. Further in-depth contextually grounded studies exploring the acceptability of such interventions are required, as well as studies exploring the cost-effectiveness of involving CHWs in cervical cancer screening activities.

**Key questions**

**What is already known?**

- Screening can help to reduce morbidity and mortality from cervical cancer.
- Community health workers (CHWs) have been proposed as one strategy to help reduce cervical cancer morbidity and mortality globally, especially in regions of the world where there are shortages of healthcare professionals to conduct screening.

**What are the new findings?**

- From the 15 studies identified and included in the review, CHWs were noted to play a role in community education and awareness raising initiatives, assisting in or conducting screening, and follow-up during the screening process.

**What do the new findings imply?**

- The use of CHWs to assist in cervical cancer screening in LMICs appears largely feasible and acceptable, although adopting participatory approaches to the design of CHW interventions may further enhance acceptability.
- Further studies evaluating the cost-effectiveness of CHWs in the delivery of integrated cervical screening and cancer care are required.

## Introduction

Cervical cancer prevention and screening has been described as one of the last frontiers of Universal Health Coverage.^1 2^ Effective vaccines now exist for human papilloma virus (HPV) (which causes the majority of cervical cancer), and screening tests and preventative treatments are available. However, access to, and provision of, screening largely depends on the presence of robust health systems, with a trained workforce and appropriate funding.

Globally, deaths from cervical cancer reflect the harsh realities of the socioeconomic disparities facing women in low- and middle-income countries (LMICs), perhaps more starkly than any other cancer. Over 85% of the 275 000 deaths each year due to cervical cancer occur in LMICs.^3^ In recognition of the increasing burden of cervical cancer in LMIC settings, the WHO published guidelines in 2013 recommending that in areas where access to a Papanicolaou test (Pap smear), cytology services and colposcopy for cervical cancer was not available, alternative evidence-based cervical screening methods could be used to screen women.^4^ These methods included HPV testing and visual inspection of the cervix by trained health workers. This policy has been adopted by some LMICs, for example in Thailand, where the Thai Ministry of Public Health recommend that women aged between 30 and 60 should be screened every 5 years by any available method.^5^

Acceptability and uptake of these interventions by women may be affected by limited knowledge of the symptoms and consequences of cervical cancer,^6 7^ lack of adequate training for self-collected vaginal specimens for HPV testing, and wider sociocultural factors.^8^ Novel methods for improving uptake and implementation of cervical screening in LMICs are required, if we are to meet the Sustainable Development Goal to reduce cancer deaths and provide universal health coverage for essential health services worldwide.

Alternative strategies are required to address the shortage of trained health workers to conduct cervical cancer screening, especially in LMIC settings. One potential solution could be the use of community health workers (CHWs). CHWs originate from the communities they serve and are uniquely placed to improve the cultural legitimacy, trust, and acceptability of cervical cancer screening interventions within their communities. They may also be trained to deliver community-based cervical ‘screen and treat’ programmes.^9–13^ A recent review by Driscoll *et al* in 2018 suggested that ‘visual inspection programme using adequately trained CHWs could help to reduce barriers and expand access to screening’ in LMICs^13^; however, no review has been undertaken to date to investigate the wider roles CHWs play in cervical cancer screening.

In this scoping review, we reviewed the existing literature for evidence of the roles of CHWs in cervical cancer screening in LMIC settings. We sought evidence to evaluate three broad areas:

- Where and how CHWs are currently deployed in cervical cancer screening in LMIC settings.
- The methods used to train and support CHWs in cervical cancer screening, including the content, duration and outcomes of training.
- The evidence on the cost-effectiveness of using CHWs to assist in cervical cancer screening.

## Methods

### Nature of review

To understand and outline the ways in which CHWs are involved in cervical cancer screening across LMICs, a systematic scoping review was conducted in September 2018. A scoping review addresses a broad exploratory research topic through outlining key concepts, types of evidence, and gaps in research following a systematic literature search.^14^ Compared with traditional systematic reviews, scoping reviews place less emphasis on quality appraisal of the included evidence.^15^ A scoping literature review was chosen for this study since it enabled us to review a broad body of literature and describe the current ways in which CHWs are involved in cervical cancer screening across a variety of different geographical contexts. We followed established guidance for conducting scoping reviews.^14 16^

### Search strategy and study selection criteria

The methodology for the scoping review was based on a previous review that was conducted by the same lead author in 2018, regarding the role of ongoing training for CHWs in LMICs.^17^ A search of the Cochrane Library, the Campbell Collaboration, the International Prospective Register of Systematic Reviews and grey literature identified no existing or scheduled reviews on the topic of CHWs and cervical cancer screening.

We designed a thorough and sensitive search strategy through developing terms for ‘Community Health Workers’ and ‘cervical cancer’ (see online supplementary table 1). Studies were manually filtered at the title and abstract screening stage, using the World Bank Group 2018 classification of economies to include those defined as LMICs.

10.1136/bmjgh-2019-001452.supp1 Supplementary data

We searched the following databases for studies published between 12th September 1978 (the date of the Alma Ata Declaration, which declared CHWs as central to primary healthcare)^18^ and September 20th 2018: Medline; Embase, AMED and Global Health via Ovid; CINAHL via Ebsco; PsychInfo; Web of Science; Scopus; ASSIA via ProQuest; British Education Index; ERIC (the full search strategy for each database is listed in online supplementary table 1). We focused on primary research or descriptive studies relevant to the research aims, and excluded letters, commentaries, opinion pieces, study protocols, policy briefings, training needs assessments and conference abstracts. We included additional non-peer-reviewed literature identified through the e-theses online service, Google Scholar, and websites of research institutions, charities, relevant government departments and international agencies involved in ear and hearing care. We also conducted a manual search of grey literature databases. Finally, we searched the reference lists of all relevant papers identified, using snowball sampling. No restrictions were placed on language.

### Inclusion and exclusion criteria

Studies were included if:

1. The primary participants of the study were CHWs. To capture all relevant literature, a wide range of search terms (over 90), based on CHW descriptions used in a previous systematic review by O’Donovan *et al* (2018),^17^ Ballard *et al* (2017),^19^ and Olaniran *et al* (2017)^20^ were used.
2. The CHWs worked in a country defined as low income or middle income according to World Bank Group 2018 classification of economies.
3. The primary aim of the study was to describe or evaluate the role of CHWs in cervical cancer screening.

Studies were excluded if:

1. The primary focus of the study was on health workers other than CHWs. For example, studies which assessed the role of medical professionals such as doctors, medical students, nurses or allied healthcare professionals, such as physician assistants, were excluded.
2. The focus of the study was on an aspect of cervical cancer not related to screening. For example, studies focusing on the role of CHWs in primary prevention strategies, such as HPV vaccination, were not included.
3. The study was not a full-text original study; articles such as commentaries, letters, review articles, policy briefs and study protocols were excluded.

Both quantitative and qualitative studies were included. Studies did not require a comparison group for inclusion.

#### Population

In this study (and consistent with agreed definitions), we defined CHWs as health workers who are members of the communities where they work, but without formal professional or paraprofessional certificated tertiary education.^21^ They should work in the community (rather than a health facility), belong to the formal health system, and perform tasks related to healthcare delivery.^21^

#### Intervention

Included studies focused on the role of CHWs in cervical cancer screening. For the purpose of this review, screening was used as an umbrella term for screening as a form of secondary prevention and could encompass any modality of screening, including Visual Inspection after application of Acetic Acid (VIA), Visual Inspection using Lugol's Iodine, Pap smear, HPV-DNA screening, or a combination of modalities.

#### Comparator

A comparator was not included.

#### Outcomes

The outcomes for our scoping review were documenting the geographical location and role for CHWs in cervical cancer screening across LMIC settings. We also were interested in the methods used to train and support CHWs, and any evidence regarding the cost-effectiveness of using CHWs to assist in cervical cancer screening.

### Study selection

Papers identified during the search were exported into EndNote 7.1 and duplicates removed. Titles and abstracts of the remaining studies were independently screened for inclusion in the study by two of the study authors (JOD and COD).

### Data extraction and analysis

Following initial screening, full texts of potentially relevant papers were independently screened by the same two authors (JOD and COD). Data were extracted and tabulated in a data charting form in a Microsoft Excel spreadsheet. The use of a data charting form has been recommended as a key stage of conducting a scoping review.^16^

Where there was disagreement regarding inclusion or exclusion from the final scoping review, the third author (SN) was consulted. Once data were transferred into the data charting form, two authors (JOD and COD) reviewed the data to identify the key focus areas for the review.

### Ethical approval

We did not seek ethical approval for this study, since this was a review of existing published literature and did not directly involve human subjects.

### Patient and public involvement

Patients were not involved in this study. A group of CHWs from Mukono, Uganda helped to review the discussion section of the paper to ensure recommendations for future work were considered appropriate from the perspective of CHW stakeholders.

## Results

### Search results

**Figure 1:**
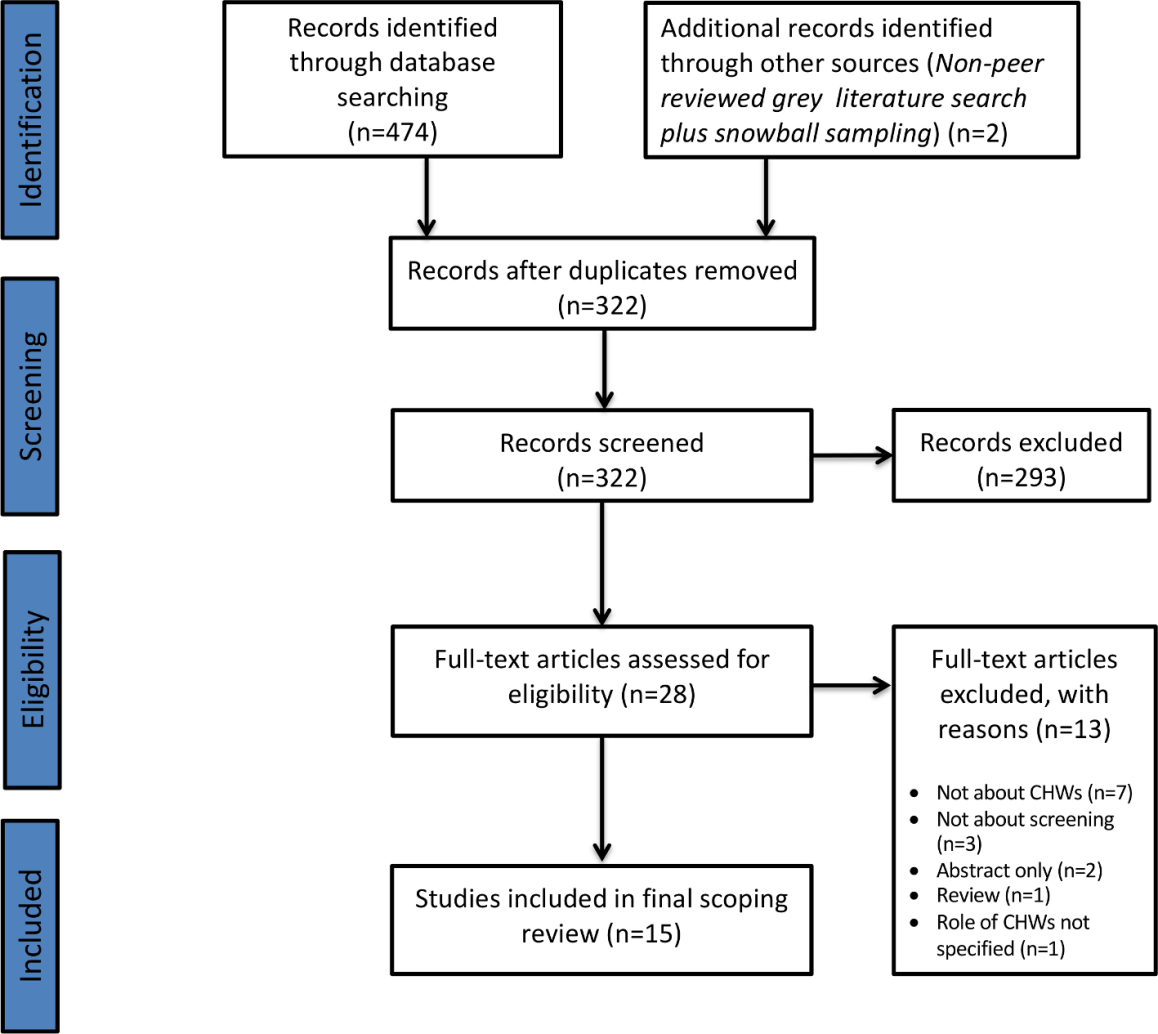
PRISMA diagram. The PRIMSA *diagram* details our search and selection process applied during the scoping review. PRISMA, Preferred Reporting Items for Systematic Reviews and Meta-Analyses.

The initial search of 11 databases and the grey literature yielded 474 articles, which was reduced to 322 after removal of duplicates (see online supplementary table 2). After the initial abstract and title screen of the 322 articles, 293 were excluded. A total of 28 studies were selected for full-text review. Following the full-text review, 13 studies were excluded based on the inclusion and exclusion criteria. Reasons for exclusion at full-text screening can be found in the Preferred Reporting Items for Systematic Reviews and Meta-Analyses flow chart (figure 1).

At the end of the screening process, 15 peer-reviewed studies remained for inclusion in the final review, of which one originated from snowball sampling, and one from the grey literature.^5 22–35^

### CHW cadres and study characteristics

**Figure 2:**
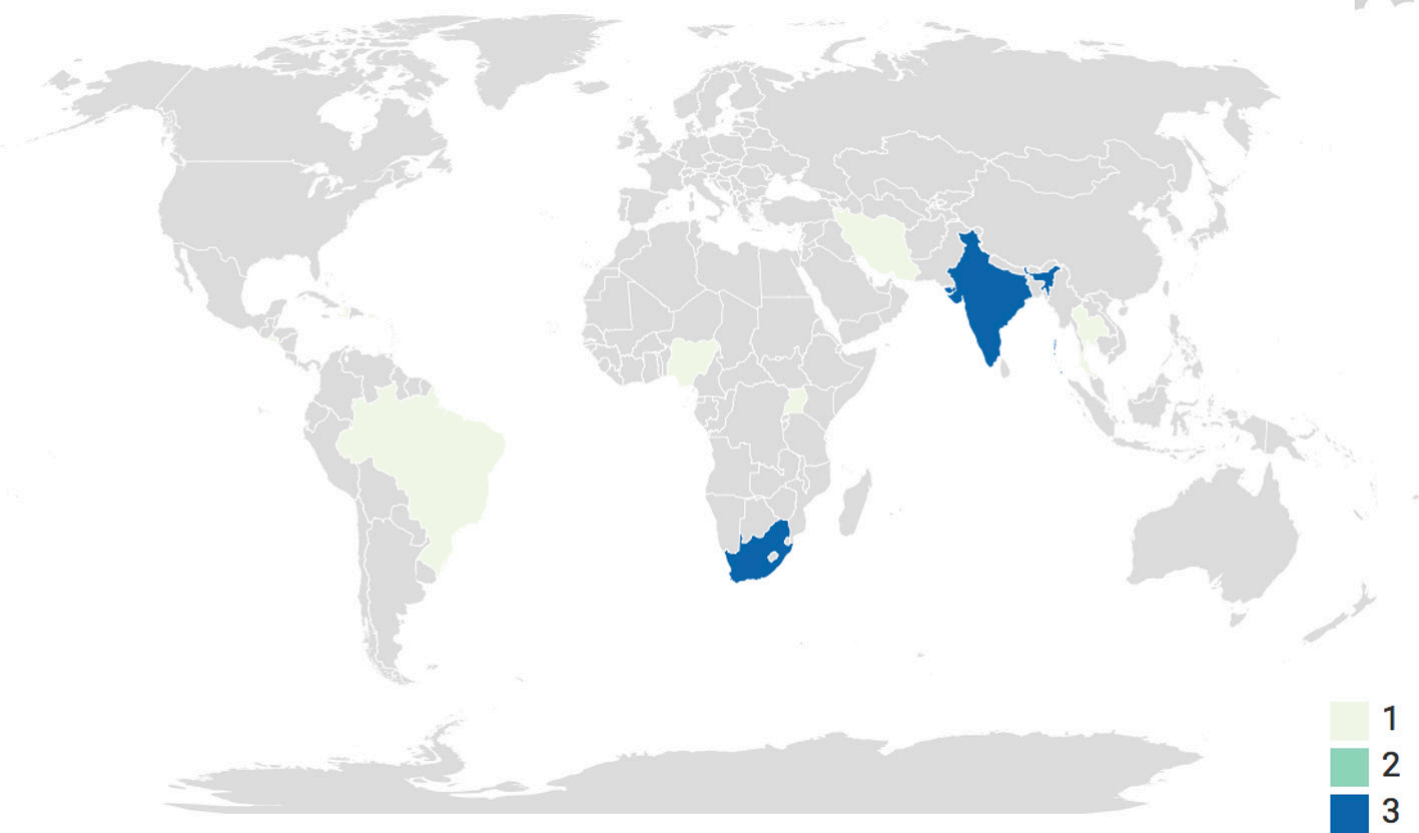
Study locations. A choropleth map highlighting the location of each study.

Across the 15 studies, which took place between 2005 and 2018, nine different terms were used to describe CHWs. South Africa (n=3) and India (n=3) were the most common country locations for the studies to take place; however globally, there was an equal geographical split between Africa (n=5), Asia (n=5) and Southern and Central America (n=5) (see figure 2).

Different modalities of screening were used across the 15 studies. Pap smears were the most common modality offered (n=6),^5 24–27 34^ followed by dual or mixed screening modalities (using a combination of Pap smears, VIA or HPV-DNA) (n=5),^23 29 32 33 35^ HPV-DNA sampling only (n=3),^22 30 31^ and VIA only (n=1).^28^

Full details of CHW cadre descriptions and study characteristics can be found in online supplementary table 3.

### Roles of CHWs

Different roles for CHWs within the screening process were described across the 15 studies (see online supplementary table 3).

The most common role for CHWs was to carry out education, outreach, or awareness raising activities (n=14).^5 22 24–35^ Home-based community outreach programmes were described in studies in El Salvador and Iran.^22 25^ In El Salvador, CHWs identified unscreened women and conducted outreach visits to their homes.^22^ The CHWs delivered an education session to women covering methods for cervical screening and treatment, as well as exploring reasons why women had not undergone screening. In Iran, CHWs were trained to deliver an educational outreach session to women on the importance of screening and how to use a self-administered Pap test. This resulted in increased cervical screening rates, from 0% to 62.85%, over a period of 2 months.^25^ It is also important to note that the majority of studies reported outreach initiatives in community settings. For futher details on specific outreach stategies, please refer to online supplementary table 3.

In five studies, CHWs played a role in the actual cervical screening process, either through performing cervical screening or in assisting specialist medical staff.^5 23 32 34 35^ In Nigeria, CHWs were trained to carry out screening using VIA under supervision, and link positive cases for cryotherapy.^23^ After 12 months of initial training, 848 women were screened by the CHWs. In all, 63 of these women were rescreened by the CHWs, with an 88.1% agreement with an expert review by a team comprised of a consultant gynaecologist, a senior resident in gynaecology and a specialist cytology nurse. Similarly, in Thailand and India, CHWs were trained to perform PAP smears and VIA screening.^5 35^

In the remaining studies, CHWs had roles in assisting the specialist medical staff who carried out the process. These included helping women undress and dress and supporting them during the screening procedure,^34^ assisting the nurse by cleaning equipment,^34^ or transporting HPV-DNA samples to the laboratory for sequencing.^32^

CHWs also had a role in the follow-up of women after cervical screening. In Peru, CHWs accompanied women who were HPV positive on screening to follow-up appointments at a clinic, with a 90% attendance rate at 6-month follow-up.^30^

### CHW training

In total, 11 studies provided details regarding how CHWs were trained to manage cervical cancer screening; however, the level of detail was variable, ranging from a brief description of the training content, to a full overview of training contents, duration, methods of assessment, and supervision (see online supplementary table 3).

Kienen *et al* (2018) provided significant detail as to how 15 CHWs in Brazil were trained over a period of 2 months to improve cervical cancer screening rates among under-screened and unscreened women aged between 25 and 64 years.^29^ In this study, four sessions regarding the theoretical knowledge of cervical cancer, behaviour change, skills development and the protection of human subjects in research were covered over a period of three days. CHWs were evaluated at the end of training using multiple choice questionnaires in order to assess their ‘objective knowledge, perceived knowledge, perceived skills, and perceived confidence across the four domains.^29^

Training evaluation was poorly documented in general. Only four studies documented how CHW training was evaluated, with pre-training and post-training written assessments being the most popular form of assessment.^29 33–35^

Four out of the 15 studies identified for inclusion in the review provided no information on the content or duration of training, who delivered training, means of evaluation, theoretical underpinnings, or supervision.^5 22 26 27^

### Financial considerations

Two studies by Goldhaber-Fiebert *et al* provided details of the cost of deploying CHWs to assist in cervical cancer screening.^26 27^ They found that CHWs in South Africa were able to successfully re-establish contact with women who missed scheduled visits for cervical cancer screening and increase their return rate.^26 27^ They provided the costs of fuel, transport, CHWs wages and other programmatic costs; however they did not conduct a formal cost analysis.

The only study to conduct a formal cost analysis was that by Mezei *et al* in 2018 in Uganda.^36^ In this study, the authors evaluated the cost-effectiveness of community-based HPV testing using self-collection kits facilitated by CHWs, with clinic-based VIA of HPV-positive women. The role of CHWs in this study was to recruit community members, teach them about how to perform self-collection of the HPV sample, and then transport the samples to a laboratory in Kampala. Using a Monte Carlo simulation model, linked to data collected from an ongoing longitudinal study in Uganda, the authors were able to project the lifetime health and economic outcomes associated with the two different techniques. They found that in all instances community-based HPV testing was more cost-effective than clinic-based VIA, with an incremental cost-effectiveness ratio ranging from $130 per years of life saved if performed once during a lifetime, to $470 if performed five times.

### Challenges of deploying CHWs to assist in cervical screening programmes

Several challenges were raised regarding the deployment of CHWs to assist in cervical cancer screening. In Thailand, Srisuwan *et al* found that many Thai women felt uncomfortable with a CHW from the same village performing a procedure they regarded as sensitive and private.^5^ This resulted in screening rates being as low as 47.3%; well below the 80% coverage target set by the government.

Other challenges involved the delivery of community-based education and awareness programmes. In the study by Colon-Lopez *et al*, the authors documented that the educational material provided to CHWs did not accurately represent the culture of the women being screened.^24^ It was hypothesised that this could negatively affect the ‘participant’s identification with programme models and influencing message processing’.^24^

Issues around long-term motivation and sustainability of such programmes were also raised as a potential challenge. For example, in the study by Colon-Lopez *et al* (2017), there was a high turnover of CHWs; although 14 CHWs were trained, only four were active at any one time, including a 4-month period where no CHWs were active.^24^ The CHWs in this particular study cited that they wished to receive higher rates of payment for conducting outreach work.

There was also a degree of fear and anxiety of women around the treatments for cervical cancer following screening, which impacted on follow-up rates of women. For example, Isaac *et al* (2012) found that of the 3182 women that were screened in India between 2009 and 2011, 36 were VIA positive and referred to a health centre for further testing and cryotherapy; however, 22 failed to attend.^28^ They cited issues such as being scared of treatment, not having symptoms therefore not wanting treatment, and husbands not wanting them to go for treatment.^28^ CHWs could therefore have a role in ensuring follow-up during the screening process.

## Discussion

The findings from this scoping review highlight the diverse range of ways CHWs have been deployed to assist in cervical cancer screening across 11 different LMICs. These ranged from community education and awareness raising initiatives, to assisting in, or conducting screening, to follow-up. Various challenges were also highlighted, including cultural acceptability, loss to follow-up and the need to ensure CHWs were adequately remunerated. Reporting of CHW training for cervical cancer screening was also highly variable, and there was a lack of evidence evaluating the cost-effectiveness of deploying CHWs to assist in screening.

The most common finding from the studies included in this review was that CHWs have a potentially useful role in sensitising women about the importance of cervical cancer screening and follow-up through educational outreach initiatives. Indeed, the role of CHWs to improve screening uptake for other disease groups, such as colorectal cancer and breast cancer, following educational outreach initiatives has been demonstrated in the USA.^37 38^ However, as several of the studies in this review suggested, sensitisation and awareness raising alone are not enough to improve screening uptake.^25 28 33^ During the design phase, screening programmes should take into account structural and cultural barriers that may negatively impact on the uptake of screening services and, where possible, address these. For example, lack of transport, discomfort with male service providers and family commitments have all been shown to be barriers that negatively influence uptake of cervical cancer screening services among women in LMICs.^39 40^

Undertaking community-based participatory research (CBPR) to facilitate the design of such programmes could be one way to help maximise the chances of a screening programmes success.^24 41 42^ By taking such an approach, potential barriers to screening uptake and follow-up care can be considered and addressed in a culturally sensitive manner.^30 43^ Indeed, CBPR approaches (which are underpinned by flexibility and a high degree of community engagement) have had success in helping to reduce disparities related to cancer screening in areas of the USA where high health disparities prevail.^44 45^ In this review, we identified key barriers, including the use of educational material which did not accurately represent the culture of the women being screened, and women being uncomfortable with CHWs from the same village conducting screening. Use of participatory approaches, such as CBPR in the design phase of cervical screening programmes, may offer potential solutions to these barriers.

We identified a lack of detail regarding the design, delivery and evaluation of training for CHWs to provide cervical cancer screening services. In particular, it was unclear if the training was evidence-based or theory-informed. In addition to developing theory-informed approaches to CHW training and evaluation of CHW training programmes, it would be important to ensure that training fits with the needs of CHWs as learners, in a culturally sensitive manner. Participatory approaches may be useful in the co-design of CHW training programmes, as it has been suggested that such approaches, which encourage deep reflection on what is being taught, can help CHWs ‘understand not only the mechanics of delivering the intervention but also the principles and theory on which it is based’.^46^ Similarly, the majority of studies which discussed the evaluation of training described the use of pre-training and post-training assessments, using either written assignments or multiple choice questions. The use of such methods of assessment does not necessarily help us to understand change in practice and behaviour of CHWs.^17^ Developing more nuanced methods of evaluation, such as the use of longitudinal measures of in-work observational assessments should be considered.

In addition, the importance of appropriate supervision and mentoring should not be underestimated. Many of the training courses detailed in this review were initial one-off courses lasting between 2 and 7 days. This could be due to the fact that many of the courses were delivered by expert clinicians, who have other commitments to attend to. It is therefore important to consider the need for ongoing training and supervision, which have been highlighted as important areas of well-functioning CHW programmes.^47^ One such approach that could be adopted to help support supervision and ongoing training could be the use of mobile technologies. Such strategies have proved successful to help support ongoing and refresher training with CHWs in the past; however, it is important to caveat that an individual context-specific assessment should be conducted prior to the implementation of such an intervention to ensure local buy-in, feasibility, and sustainability.

Finally, from the studies identified, financial details regarding the deployment of CHWs to assist in cervical cancer screening was mentioned in three studies, only one of which was a formal cost analysis.^26 27 32^ Given that a recent cost-effectiveness analysis conducted by Mezei *et al* (2017) concluded that policy makers should explore the role of HPV testing with self-collection of samples as the most cost-effective strategy,^48^ future studies involving CHWs to facilitate in such a role should be considered.

Regarding study limitations, it is likely there are other existing and ongoing initiatives utilising CHWs to play a role in screening for cervical cancer that have not been reported in this study since they have not been formally published. Many of the studies focused primarily on eliciting the views of women in the target screening population, rather than CHWs themselves. Therefore, there is a need for more studies to explore the perspectives of CHWs regarding their role in cervical cancer screening. It is also important to note that we did not perform a quality assessment of studies; however, this in line with the widely accepted guidelines for conducting a scoping review.^15 16^ This means, however, that we can only highlight the existing evidence base, and not make recommendations based on the quality of evidence. Finally, there was heterogeneity of the studies included in the final review in terms of scope and methodology. Many of the studies evaluated the outcomes of entire programmes in which CHWs played a role in facilitating or conducting screening, which is not the same as specifically evaluating the additive role of CHWs. The evidence base is therefore relatively narrow, and led to more of a narrative, than systematic, description of findings and roles of CHWs in cervical cancer screening.

## Conclusion

In conclusion, from the limited number of studies available, the use of CHWs to assist in cervical cancer screening in LMICs appears largely feasible and acceptable. CHWs currently have an important role in cervical cancer screening mainly through community education, outreach, and awareness activities. Several gaps were also identified in the existing literature, including a need for a more studies to explore training and ongoing support for CHWs, as well as the financial implications of deploying CHWs to assist in cervical cancer screening. Finally, an important caveat is that CHWs cannot be seen as a silver bullet solution to address the burden of cervical cancer in LMICs. Although they may have a potentially important role in screening activities, it is essential that policy makers and governments ensure adequate provision of secondary and tertiary services for those women identified through screening activities as needing specialist management.
