# Supplementary material for: The role of community health workers in cervical cancer screening in low-income and middle-income countries: a systematic scoping review of the literature
Source: BMJ Glob Health. 2019 May 13;4(3):e001452. doi: 10.1136/bmjgh-2019-001452 (PMC6528769; doi:10.1136/bmjgh-2019-001452)
Supplement: Supplementary data [file bmjgh-2019-001452supp001.pdf]

Supplementary Material.

Table 1. Database search strategies, including search terms.

Database(s)	Search strategy
**AMED (Allied and Complementary Medicine) 1985 to September 2018, Embase 1974 to present, Global Health 1973 to 2018 Week 37, Medline 1946 to present, PsycINFO 1806 to present**	(*accompagnateurs* OR *activista* OR *"Agente comunitario de salud"* OR *"Agente comunitario de saude"* OR *"Agent de Sante Communautaire"* OR *"Agentes Polivalentes Elementares"* OR *"Allied health personnel"* OR *anganwadi* OR *"Animatrice de Sante Maternelle"* OR *"Auxiliary health worker"* OR *"Barangay health worker"* OR *"Barefoot doctor"* OR *"Basic health worker"* OR *behvarz* OR *binome* OR *brigadista* OR *"Care Facilitator"* OR *"Colaborador voluntario"* OR *"Community based reproductive health agents"* OR *"Community assistant"* OR *"Community care worker"* OR *"Community development worker"* OR *"Community drug distributor"* OR *"Community health representative"* OR *"Community health advocate"* OR *"Community health agent"* OR *"Community health aide"* OR "Community health educator" OR *"community health assistant"* OR "Community health educator" OR *"Community health promoter"* OR *"Community health volunteer"* OR *"Community health worker"* OR *"Community health extension worker"* OR *"Community nutrition worker"* OR *"Community mobilizer"* OR *"Community resource person"* OR *"Community reproductive health worker"* OR *"Community support worker"* OR *"Community volunteer"* OR *"Community-based worker"* OR *"Female community health volunteer"* OR *"Female multipurpose health worker"* OR *"Frontline health worker"* OR *"Health extension worker"* OR *"Health and nutrition worker"* OR *"Health service assistant"* OR *"Health surveillance assistant"* OR *"Health promoter"* OR *"Home health aide"* OR *"Health Volunteer"* OR *"Home visitor"* OR *kader* OR *"Lady health worker"* OR *"Lay health visitor"* OR *"Lay health worker"* OR *"Link worker"* OR *"Maternal and child health promotion worker"* OR *"Maternal and child health worker"* OR *"Mental health worker"* OR *monitira* OR *"Mother coordinator"* OR *"Nutrition volunteer"* OR *"Nutrition worker"* OR *"Outreach educator"* OR *"Paramedical worker"* OR *"Patent Medicine Vendor"* OR *"Peer volunteer"* OR *"Postnatal support worker"* OR *"Primary health care worker"* OR *promotora* OR *raedat* OR *"Relais communautaire"* OR *"Rural health motivator"* OR *"Rural health worker"* OR *"Rural health care worker"* OR *"Saksham sahaya"* OR *sevika* OR *"Shastho karmis"* OR *"Shastho shebika"* OR *visitadora* OR *"Village drug-kit manager"* OR *"Village Health Guide"* OR *"Village health helper"* OR *"Village health worker"* OR *"Village health committee"* OR *"Village health team"* OR *"Voluntary worker"* OR *"Volunteer health worker"* OR *"Voluntary Community Health Worker"* OR *"Women group leader"*).mp [mp=ab, hw, ti, tn, ot, dm, mf, dv, kw, fx, dq, bt, id, cc, nm,

	kf, px, rx, ui, sy, tc, tm]<br><br>AND<br><br>*("cervical screen*" OR "cervical cancer" OR "cervical neoplasm" OR "cervical malignan*" OR "colposcopy" OR "HPV DNA" OR "PAP smear" OR "Papanicolaou" OR "direct visualisation" OR "liquid cytology" OR "visual inspection")*mp. [mp=ab, hw, ti, tn, ot, dm, mf, dv, kw, fx, dq, bt, id, cc, nm, kf, px, rx, ui, sy, tc, tm]
**ASSIA (via Proquest)**	(accompagnateurs OR activista OR "Agente comunitario de salud" OR "Agente comunitario de saude" OR "Agent de Sante Communautaire" OR "Agentes Polivalentes Elementares" OR "Allied health personnel" OR anganwadi OR "Animatrice de Sante Maternelle" OR "Auxiliary health worker" OR "Barangay health worker" OR "Barefoot doctor" OR "Basic health worker" OR behvarz OR binome OR brigadista OR "Care Facilitator" OR "Colaborador voluntario" OR "Community based reproductive health agents" OR "Community assistant" OR "Community care worker" OR "Community development worker" OR "Community drug distributor" OR "Community health representative" OR "Community health advocate" OR "Community health agent" OR "Community health aide" OR "Community health educator" OR "community health assistant" OR "Community health educator" OR "Community health promoter" OR "Community health volunteer" OR "Community health worker" OR "Community health extension worker" OR "Community nutrition worker" OR "Community mobilizer" OR "Community resource person" OR "Community reproductive health worker" OR "Community support worker" OR "Community volunteer" OR "Community-based worker" OR "Female community health volunteer" OR "Female multipurpose health worker" OR "Frontline health worker" OR "Health extension worker" OR "Health and nutrition worker" OR "Health service assistant" OR "Health surveillance assistant" OR "Health promoter" OR "Home health aide" OR "Health Volunteer" OR "Home visitor" OR kader OR "Lady health worker" OR "Lay health visitor" OR "Lay health worker" OR "Link worker" OR "Maternal and child health promotion worker" OR "Maternal and child health worker" OR "Mental health worker" OR monitira OR "Mother coordinator" OR "Nutrition volunteer" OR "Nutrition worker" OR "Outreach educator" OR "Paramedical worker" OR "Patent Medicine Vendor" OR "Peer volunteer" OR "Postnatal support worker" OR "Primary health care worker" OR promotora OR raedat OR "Relais communautaire" OR "Rural health motivator" OR "Rural health worker" OR "Rural health care worker" OR "Saksham sahaya" OR sevika OR "Shastho karmis" OR "Shastho shebika" OR visitadora OR "Village drug-kit manager" OR "Village Health Guide" OR "Village health helper" OR "Village health worker" OR "Village health committee" OR "Village health team" OR "Voluntary worker" OR

	"Volunteer health worker" OR "Voluntary Community Health Worker" OR "Women group leader")<br><br>AND<br><br>*("cervical screen*" OR "cervical cancer" OR "cervical neoplasm" OR "cervical malignan*" OR "colposcopy" OR "HPV DNA" OR "PAP smear" OR "Papanicolaou" OR "direct visualisation" OR "liquid cytology" OR "visual inspection")*
**SCOPUS**	TITLE-ABS-KEY (*accompagnateurs* OR *activista* OR *"Agente comunitario de salud"* OR *"Agente comunitario de saude"* OR *"Agent de Sante Communautaire"* OR *"Agentes Polivalentes Elementares"* OR *"Allied health personnel"* OR *anganwadi* OR *"Animatrice de Sante Maternelle"* OR *"Auxiliary health worker"* OR *"Barangay health worker"* OR *"Barefoot doctor"* OR *"Basic health worker"* OR *behvarz* OR *binome* OR *brigadista* OR *"Care Facilitator"* OR *"Colaborador voluntario"* OR *"Community based reproductive health agents"* OR *"Community assistant"* OR *"Community care worker"* OR *"Community development worker"* OR *"Community drug distributor"* OR *"Community health representative"* OR *"Community health advocate"* OR *"Community health agent"* OR *"Community health aide"* OR *"community health assistant"* OR "Community health educator" OR *"Community health promoter"* OR *"Community health volunteer"* OR *"Community health worker"* OR *"Community health extension worker"* OR *"Community nutrition worker"* OR *"Community mobilizer"* OR *"Community resource person"* OR *"Community reproductive health worker"* OR *"Community support worker"* OR *"Community volunteer"* OR *"Community-based worker"* OR *"Female community health volunteer"* OR *"Female multipurpose health worker"* OR *"Frontline health worker"* OR *"Health extension worker"* OR *"Health and nutrition worker"* OR *"Health service assistant"* OR *"Health surveillance assistant"* OR *"Health promoter"* OR *"Home health aide"* OR *"Health Volunteer"* OR *"Home visitor"* OR *kader* OR *"Lady health worker"* OR *"Lay health visitor"* OR *"Lay health worker"* OR *"Link worker"* OR *"Maternal and child health promotion worker"* OR *"Maternal and child health worker"* OR *"Mental health worker"* OR *monitira* OR *"Mother coordinator"* OR *"Nutrition volunteer"* OR *"Nutrition worker"* OR *"Outreach educator"* OR *"Paramedical worker"* OR *"Patent Medicine Vendor"* OR *"Peer volunteer"* OR *"Postnatal support worker"* OR *"Primary health care worker"* OR *promotora* OR *raedat* OR *"Relais communautaire"* OR *"Rural health motivator"* OR *"Rural health worker"* OR *"Rural health care worker"* OR *"Saksham sahaya"* OR *sevika* OR *"Shastho karmis"* OR *"Shastho shebika"* OR *visitadora* OR *"Village drug-kit manager"* OR *"Village Health Guide"* OR *"Village health helper"* OR *"Village health worker"* OR *"Village health committee"* OR *"Village health*

	team" OR *"Voluntary worker"* OR *"Volunteer health worker"* OR *"Voluntary Community Health Worker"* OR *"Women group leader"*) = 32,145 hits<br><br>**AND**<br><br>TITLE-ABS-KEY (*"cervical screen*" OR "cervical cancer" OR "cervical neoplasm" OR "cervical malignan*" OR "colposcopy" OR "HPV DNA" OR "PAP smear" OR "Papanicolaou" OR "direct visualisation" OR "liquid cytology" OR "visual inspection")*
**Web of Science**	(TOPIC: ((accompagnateur OR activists) OR "Agente comunitario de salud") OR "Agente comunitario de saude") OR "Agent de Sante Communautaire") OR "Agentes Polivalentes Elementares") OR "Allied health personnel") OR anganwadi) OR "Animatrice de Sante Maternelle") OR "Auxiliary health worker") OR "Barangay health worker") OR "Barefoot doctor") OR "Basic health worker") OR bednarz) OR binomen) OR brigadistas) OR "Care Facilitator") OR "Colaborador voluntario") OR "Community based reproductive health agents") OR "Community assistant") OR "Community care worker") OR "Community development worker") OR "Community drug distributor") OR "Community health representative") OR "Community health advocate") OR "Community health agent") OR "Community health aide") OR "community health assistant") OR Community health educator) OR "Community health promoter") OR "Community health volunteer") OR "Community health worker") OR "Community health extension worker") OR "Community nutrition worker") OR "Community mobilizer") OR "Community resource person") OR "Community reproductive health worker") OR "Community support worker") OR "Community volunteer") OR "Community-based worker") OR "Female community health volunteer") OR "Female multipurpose health worker") OR "Frontline health worker") OR "Health extension worker") OR "Health and nutrition worker") OR "Health service assistant") OR "Health surveillance assistant") OR "Health promoter") OR "Home health aide") OR "Health Volunteer") OR "Home visitor") OR kader) OR "Lady health worker") OR "Lay health visitor") OR "Lay health worker") OR "Link worker") OR "Maternal and child health promotion worker") OR "Maternal and child health worker") OR "Mental health worker") OR monitra) OR "Mother coordinator") OR "Nutrition volunteer") OR "Nutrition worker") OR "Outreach educator") OR "Paramedical worker") OR "Patent Medicine Vendor") OR "Peer volunteer") OR "Postnatal support worker") OR "Primary health care worker") OR promotora) OR rawdat) OR "Relais communautaire") OR "Rural health motivator") OR "Rural health worker") OR "Rural health care worker") OR "Saksham sahaya") OR sevina) OR "Shastho karmis") OR "Shastho shebika") OR visitadoras) OR "Village drug-kit manager") OR

	"Village Health Guide") OR "Village health helper") OR "Village health worker") OR "Village health committee") OR "Village health team") OR "Voluntary worker") OR "Volunteer health worker") OR "Voluntary Community Health Worker") OR "Women group leader") *AND*<br><br>AND<br><br>TOPIC: *"cervical screen*" OR "cervical cancer" OR "cervical neoplasm" OR "cervical malignan*" OR "colposcopy" OR "HPV DNA" OR "PAP smear" OR "Papanicolaou" OR "direct visualisation" OR "liquid cytology" OR "visual inspection"*
**CINAHL, BEI and ERIC**	*accompagnateurs* OR *activista* OR *"Agente comunitario de salud"* OR *"Agente comunitario de saude"* OR *"Agent de Sante Communautaire"* OR *"Agentes Polivalentes Elementares"* OR *"Allied health personnel"* OR *anganwadi* OR *"Animatrice de Sante Maternelle"* OR *"Auxiliary health worker"* OR *"Barangay health worker"* OR *"Barefoot doctor"* OR *"Basic health worker"* OR *behvarz* OR *binome* OR *brigadista* OR *"Care Facilitator"* OR *"Colaborador voluntario"* OR *"Community based reproductive health agents"* OR *"Community assistant"* OR *"Community care worker"* OR *"Community development worker"* OR *"Community drug distributor"* OR *"Community health representative"* OR *"Community health advocate"* OR *"Community health agent"* OR *"Community health aide"* OR *"community health assistant"* OR "Community health educator" OR *"Community health promoter"* OR *"Community health volunteer"* OR *"Community health worker"* OR *"Community health extension worker"* OR *"Community nutrition worker"* OR *"Community mobilizer"* OR *"Community resource person"* OR *"Community reproductive health worker"* OR *"Community support worker"* OR *"Community volunteer"* OR *"Community-based worker"* OR *"Female community health volunteer"* OR *"Female multipurpose health worker"* OR *"Frontline health worker"* OR *"Health extension worker"* OR *"Health and nutrition worker"* OR *"Health service assistant"* OR *"Health surveillance assistant"* OR *"Health promoter"* OR *"Home health aide"* OR *"Health Volunteer"* OR *"Home visitor"* OR *kader* OR *"Lady health worker"* OR *"Lay health visitor"* OR *"Lay health worker"* OR *"Link worker"* OR *"Maternal and child health promotion worker"* OR *"Maternal and child health worker"* OR *"Mental health worker"* OR *monitira* OR *"Mother coordinator"* OR *"Nutrition volunteer"* OR *"Nutrition worker"* OR *"Outreach educator"* OR *"Paramedical worker"* OR *"Patent Medicine Vendor"* OR *"Peer volunteer"* OR *"Postnatal support worker"* OR *"Primary health care worker"* OR *promotora* OR *raedat* OR *"Relais communautaire"* OR *"Rural health motivator"* OR *"Rural health worker"* OR *"Rural health care worker"* OR *"Saksham sahaya"* OR *sevika* OR *"Shastho karmis"* OR *"Shastho*

	shebika" OR *visitadora* OR *"Village drug-kit manager"* OR *"Village Health Guide"* OR *"Village health helper"* OR *"Village health worker"* OR *"Village health committee"* OR *"Village health team"* OR *"Voluntary worker"* OR *"Volunteer health worker"* OR *"Voluntary Community Health Worker"* OR *"Women group leader"*<br><br>AND<br><br>*"cervical screen*" OR "cervical cancer" OR "cervical neoplasm" OR "cervical malignan*" OR "colposcopy" OR "HPV DNA" OR "PAP smear" OR "Papanicolaou" OR "direct visualisation" OR "liquid cytology" OR "visual inspection"*

Legend: Search terms for each database searched.

Table 2. Results from individual database searches.

**Database**	**Number of hits**
Medline, Embase, Global Health, PsychInfo and AMED via (OVID)	59
Scopus	175
Web of Science	83
ASSIA via ProQuest	95
BEI via EBSCO	2
ERIC via EBSCO	4
CINAHL via EBSCO	56
Grey literature and snowballing	10

Legend. The results from individual database searches, including the number of hits and the supplementary material page numbers where the search strategies can be found.

Table 3. Summary table of included studies.

Author	Study Title	Year	Country and region	Name given to CHW, number involved in study and cadre description	Purpose and duration of study, role of CHW relevant to cervical cancer screening and screening modality used	Training and supervision	Results/Key findings	Financial details or Cost analysis
Alfaro et al.	Factors affecting attendance to cervical cancer screening among women in the Paracentral Region of El Salvador: a nested study within the CAPE HPV screening program	2015	El Salvador, Central America	**Name:** Health Promoters<br><br>**Number:** No details<br><br>**Description:** Local employees of the MoH who reside in the communities in which they work and promote preventive health initiatives by providing education and counselling.	**Purpose:** To identify factors affecting public sector HPV DNA-based cervical cancer screening participation in El Salvador.<br><br>**Duration:** 6 months<br><br>**Role of CHW:** Health promoters used health unit cytology registries to identify women not screened within the past 3 years and visited them in their home to promote screening. They recruited them to the study, and presented the 409 women who enrolled in the study with an educational session regarding HPV, cervical cancer,	**Content:** No details<br><br>**Duration:** No details<br><br>**Person or group responsible for delivering training:** No details<br><br>**Theories used:** No details<br><br>**Assessment of training:** No details<br><br>**Supervision:** No details	410 women were selected to participate in the study to receive the intervention.<br><br>Adherence to screening was similar between women participating in the Screening Adherence study (88.0% of 409) compared to women not participating (85.5% of 1487; p = 0.23)<br><br>Women who had previously not undergone screening in the past three years cited factors including feeling that CC screening was painful (61.4%), uncomfortable or embarrassing (64.3%), and women did not like having to see male providers (71.4%). Many women were unsure of the purpose of a Pap test (61.4%), did	No details

				**Remuneration:** No details	screening methods, HPV testing, self-sampling and provider-collected sampling, interpreting HPV results, and possible treatments.<br><br>**Screening modality:** HPV-DNA screening		not recall any suggestions around screening from their providers (65.7%), or believed that cervical cancer screening was unnecessary (65.7%).	
Awoulde et al.	Screen and Triage by Community Extension Workers to Facilitate Screen and Treat: Task-Sharing Strategy to Achieve Universal Coverage for Cervical Cancer Screening in Nigeria	2018	Nigeria, West Africa	**Name:** Community Health Extension Workers (CHEWs)<br><br>**Number:** 37<br><br>**Description:** Workers involved in task shifting and task sharing, CHEWs complement the workforce, especially at primary health care (PHC) centres and	**Purpose:** To improve knowledge and practice skills of CHEWs in a rural community of Oyo state, Nigeria, through training and participatory supervision to screen for cervical cancer using visual inspection with acetic acid and link positive cases for treatment with cryotherapy.<br><br>**Duration:** 12 months<br><br>**Role of CHW:** To provide quality cervical cancer screening services and increase population coverage of the service	**Content:** Focused on the principles of Visual Inspection with Acetic Acid. CHEWs were trained using didactic lectures, picture training of normal and abnormal VIA test results, and classroom pelvic model practice CHEWs also had hands-on training performing and interpreting VIA tests, the appropriate referral system	After the training, cervical cancer and its prevention knowledge improved from 52.4% before training to 91.5% immediate after training. Over 12 months, 950 eligible women were screened, of whom 848 (89.3%) were screened by CHEWs and CHOs. Of the 63 rescreened by CHEWs and CHOs (data grouped), and nurses, 88.1% and 92.3%, respectively, agreed with expert team review, with κ statistics of 0.76 and 0.84, respectively.	No details

				in rural communities.<br><br>**Remunerati on:** No details	in the local government area. The CHEWs were trained to perform VIA and PAP smears.<br><br>**Screening modality:** VIA and PAP smear	to the general hospital that served as the hub for the PHCs, and appropriate evaluation and treatment of cases of cervical lesions using cryotherapy for eligible cases and referral for other cases.<br><br>**Duration:** 5 days<br><br>**Person or group responsible for delivering training:** A consultant gynaecologist, a senior resident in obstetrics and gynaecology, and a cytology nurse<br><br>**Theories used:** No details		

						**Assessment of training:** No details<br><br>**Supervision:** Yes. The CHEWs received one to two monthly supervisory site visits throughout the study.		

Colon-Lopez et al.	Community-Academic Partnership to implement a Breast and Cervical Cancer screening education program in Puerto Rico	2017	Puerto Rico, Central America	Lay Health Workers (LHWs)<br><br>**Number:** 2<br><br>**Description:** LHWs are typically respected community members educate peers in a culturally appropriate manner. They normally live in the area where they conduct outreach.<br><br>**Remuneration:** No details	**Purpose:** To describe how a community-academic partnership between Taller Salud Inc., a community-based organization, and the Puerto Rico Community Cancer Control Outreach Program of the University of Puerto Rico was crucial in the adaptation and implementation of Cultivando La Salud (CLS), an evidence-based educational outreach program designed to increase breast and cervical cancer screening among Hispanic women living in Puerto Rico.<br><br>**Duration:** 14 months<br><br>**Role of CHW:** LHWs contacted women to set up a face-to- face session at the participant's home to conduct a cervical cancer awareness	**Content:** The role of LHW, strategies for reaching women, breast and cervical cancer epidemiology and screening, teaching methods, and practices sessions using peer-to-peer training models.<br><br>**Duration:** 2 days<br><br>**Person or group responsible for delivering training:** National Center for Farmworker Health<br><br>**Theories used:** No details<br><br>**Assessment of training:** No details	LHWs visited 3,665 households and 2,019 women were approached. 501 were eligible to take part in the study. 142 (32%) were non-adherent to cervical cancer screening. 444 women were recruited and 48% received an education session.<br><br>There was a high turnover of LHWs. 14 LHWs were trained to deliver intervention, with an average of 4 active LHWs at a time. There was a 4-month period with no LHWs. The FDG at the end revealed that LHWs wished to receive higher compensation. There was high overall acceptability of the intervention. In addition 90% of the women said they enjoyed the interaction they had with the LHW during the intervention discussion. Further, during the post-intervention follow up, some women indicated that the flipchart's images used during the education session did not represent	No details

					educational session (45-90 minutes). They covered the following topics: cancer, breast/ cervical cancer, risk factors, early cancer detection relevancy and HPV vaccination, mammography/Pap test, clinical breast exam, and communication with physician. They also included testimonials of women who underwent breast/cervical cancer screening and to identify and address barriers against screening test. At the end of each session, LHWs provided women with information about local low–cost or free breast and cervical cancer screening services<br><br>**Screening modality:** PAP smear	**Supervision:** No details	the target community culture and which could affect participant's identification with program models and influencing message processing. The study demonstrated a clear need to establish roles and responsibilities a priori.	
Ghahremani et	Investigation of the	2016	Iran, Middle-	**Name:** Health	**Purpose:** To describe the effect of	**Content:** Introduction to	No women had carried out a PAP smear test before the	No details

al.	role of training health volunteers in promoting pap smear test use among Iranian women based on the protection motivation theory		East	Volunteers (HVs)<br><br>**Number:** 60<br><br>**Description:** HVs are socially accepted among women in a neighbourhood, have spare time, and are motivated. Their role is to improve and delivery of healthcare services and help individuals in the community to live healthier.<br><br>**Remuneration:** No details	educational interventions implemented by health volunteers based on protection motivation theory (PMT) on promoting self-administered PAP test use among women in Iran.<br><br>**Duration:** 2 months<br><br>**Role of CHW:** HVs recruited women into the study, delivered an education session and trained them in how to use the PAP smear.<br><br>**Screening modality:** Self-administered PAP test	cervical cancer, signs and risk factors, complications and effects on daily life, importance of PAP test in early detection and treatment of cervical cancer, method of sampling, and places for performing PAP test.<br><br>The training was delivered through PowerPoint slide shows, educational pamphlets and booklets.<br><br>**Duration:** Three 120-minute training sessions<br><br>**Person or group responsible for**	study.<br><br>After the intervention 62.85% of women in the intervention group and 5.71% of women in the control group.<br><br>The women not carrying out PAP test cited high cost as a reason for not undertaking it. This therefore needs to be costed if the poorest members of the population are to be reached.	

						**delivering training:** No details<br><br>**Theories used:** No details<br><br>**Assessment of training:** No details<br><br>**Supervision:** No details		
Goldhaber-Fiebert et al.	The costs of reducing loss to follow-up in South African cervical cancer screening	2005	South Africa, Africa	**Name:** Community Health Workers (CHWs)<br><br>**Number:** No details<br><br>**Description:** No cadre description<br><br>**Remuneration:** 4000 South African Rand per month	**Purpose:** The aim of this study was to quantify the resources used in re-establishing contact with women who missed their scheduled cervical cancer screening visits and to assess the success of this effort in reducing loss to follow-up. Follow up was measured at 6, 12 and 24 months<br><br>**Duration:** 36 months<br><br>**Role of CHW:** The focus of the role of CHWs in this study was to reduce loss to	**Content:** No details<br><br>**Duration:** No details<br><br>**Person or group responsible for delivering training:** No details<br><br>**Theories used:** No details<br><br>**Assessment of training:** No details<br><br>**Supervision:**	CHW contact with women who missed scheduled visits increased their return rate. Costs were estimated of using CHWs to conduct the follow up and all costs were taken into account. E.g. Fuel, transport, wages compared to South African health worker wage scales. In total, CHWs conducted approximately 3,200 visits in 530 trips during 2003. Per-woman screened costs ranged from 8.59–12.62 Rand, 20.65–24.89 Rand, and 39.42–40.45 Rand for 6, 12, and 24-month appointments respectively. The costs of the CHW intervention were	Yes. Cost per home visit. Cost per woman screened.

					follow up during screening programmes. CHWs visited women in their homes if they have missed appointments for screening.<br><br>**Screening modality:** PAP smear	No details	substantial when considered in the con- text of the total per-woman cost of cervical cancer screen- ing. The CHW home visit costs were 8–15%, 15–29%, and 25–47% of the total per-woman screened cost for 6, 12, and 24-month visits	
Goldhaber-Fiebert et al.	Program Spending to Increase Adherence: South African Cervical Cancer Screening	2009	South Africa, Africa	**Name:** Community Health Workers<br><br>**Number:** No details<br><br>**Description:** No cadre description<br><br>**Remuneration:** 4000 South African Rand per month.	**Purpose:** To describe the relationship between investment in community health worker (CHW) home visits and increased attendance at cervical cancer screening appointments in Cape Town, South Africa.<br><br>**Duration:** 12 months<br><br>**Role of CHW:** The role of the CHWs drive through the community making home visits to reschedule appointments and encourage attendance, with CHW visits repeated until return is achieved or further	**Content:** No details<br><br>**Duration:** No details<br><br>**Person or group responsible for delivering training:** No details<br><br>**Theories used:** No details<br><br>**Assessment of training:** No details<br><br>**Supervision:** No details	Increased adherence was noted after each subsequent CHW visit. The costs of making the CHW visits was based on resource use including both personnel time and vehicle-related expenses valued in 2004 Rand. The CHW program cost R194,018, with 1,576 additional appointments attended. Adherence increased from 74% to 90%; 55% to 87%; 48% to 77%; and 56% to 80% for 6-, 12-, 24-, and 36-month appointments. Average per-woman costs increased by R14–R47. The majority of this increase occurred with the first 2 CHW visits (90%, 83%, 74%, and 77%; additional cost: R12–R26)	Yes.

					participation is declined<br><br>**Screening modality:** PAP smear			
Isaac et al.	Translating Evidence into Practice in Low Resource Settings: Cervical Cancer Screening Tests are Only Part of the Solution in Rural India	2012	India, Asia	**Name:** Self-help women group leaders<br><br>**Number:** 1000<br><br>**Description:** No cadre description<br><br>**Remuneration:** No details	**Purpose:** To describe a single- visit approach for cervical cancer screening using the VIA test with follow-up cryotherapy at the secondary level Community Health Centre.<br><br>**Duration:** 36 months<br><br>**Role of CHW:** The CHWs role was to educate and inform the women about the screening programme.<br><br>**Screening modality:** VIA	**Content:** CHWs were taught about causes and background of cervical cancer as well as on treatment modalities available<br><br>**Duration:** 4 months<br><br>**Person or group responsible for delivering training:** No details<br><br>**Theories used:** No details<br><br>**Assessment of training:** No details<br><br>**Supervision:** No details	Between April 2009 to October 2011, of the estimated 20,000 eligible women, 3,182 were screened (16%). 36 were VIA positive and referred to the health centre. 22 however failed to attend for further testing and cryotherapy. They cited issue such as not having symptoms therefore not wanting treatment (n=2), being scared of treatment (n=5), husbands not wishing them to go for treatment (n=2).<br><br>CHWs could therefore have had a potential role in following up these non-attenders who were positive on screening. Increasing community awareness is therefore important. Unfortunately there was a lack of detail in this paper with regards to how this was optimised or done.	No details

							There was no qualitative feedback from community members.	
Kienen et al.	Cervical Cancer Screening among Underscreened and Unscreened Brazilian Women: Training Community Health Workers to be Agents of Change	2018	Brazil, South America	**Name:** Community Health Workers (CHWs)<br><br>**Number:** 15<br><br>**Description:** CHWs are specifically placed to reach individuals who have not been reached through the programs offered in health clinics. Because they are considered trusted members of the targeted community, they can provide effective	**Purpose:** To describe the implementation and evaluation of a training model for CHWs to improve cervical cancer screening rates in Brazil.<br><br>**Duration:** 2 months<br><br>**Role of CHW:** The role of the CHW was to sensitise and advocate for women in the local community to undergo cervical screening.<br><br>**Screening modality:** Randomised to one of three groups; Pap test only, self-collection for HPV testing, and choice between Pap test and self-collection for HPV testing	**Content:** Four sessions focussing on knowledge about cervical cancer, knowledge regarding behaviour change, skills development, and knowledge and skills regarding the protection of human subjects in research were covered, because CHWs would be consenting participants to take part in the research study. There was also a focus on behavioural strategies to promote behaviour change.	Objective knowledge about cervical cancer, behaviour change, and the protection of human subjects in research increased significantly between pre-training and post-training as well as their perceived knowledge about behaviour change and cervical cancer. There were no significant changes in their perceived skills to promote behaviour change and motivate women to be screened for cervical cancer.	No details

				social support, which leads to increased access to care, especially in underserved populations. With adequate training, CHWs can actively engage in interventions that aim to promote behaviour change, including cervical cancer screening". In this study CHWs had to meet the following criteria: must be at least 18 years of age, a woman, and responsible		**Duration:** 3 days<br><br>**Person or group responsible for delivering training:** No details<br><br>**Theories used:** Two - the trans-theoretical model and the health belief model.<br><br>**Assessment of training:** Pre-test and post-test questionnaires were administered to evaluate changes in four key components: objective knowledge, perceived knowledge, perceived skills, and perceived		

				for a geographic area within the BHU.<br><br>**Remuneration:** No details		confidence. In addition to demographic information, the assessments consisted of a set of 22 multiple choice knowledge questions and 12 Likert scale questions pertaining to these four key components.<br><br>**Supervision:** No details		
Levinson et al.	The Peru Cervical Cancer Prevention Study (PERCAPS): Community Based Participatory Research in Manchay, Peru	2013	Peru, South America	**Name:** Promoteras<br><br>**Number:** 10<br><br>**Description:** No cadre description<br><br>**Remuneration:** No details	**Purpose:** To evaluate the utility of Community Based Participatory Research techniques in a mother-child screen/treat and vaccinate program for cervical cancer in Manchay, Peru.<br><br>**Duration:** 6 months<br><br>**Role of CHW:** CHWs located each HPV positive woman	**Content:** 1) education about HPV, cervical cancer, and the interventions for the study, 2) the design of the implementation plan by the CHWs (how to advertise, recruit, register, collect and report results), and 3) introduction to	4 main results: 1. CHWs mainly used a door-to-door strategy for sampling which they decided upon as part of the CBPR approach (although they questioned whether or not a door to door strategy would work at scale). 2. Forms were filled in correctly by CHWs 3. High participation (323 adults were registered, and 312 specimens (96.6%) were collected.) 4. Patients were satisfied with the door-to door model	No details

					(an instruction form for collection of the HPV self-sample was provided to each participant) and accompanied them for their treatment and 6 month follow-up appointments. They also planned to visit each participant's home or workplace for delivery of results.<br><br>**Screening modality:** HPV-DNA self-sampling	the standardized study forms created by the research team<br><br>**Duration:** 3 days<br><br>**Person or group responsible for delivering training:** No details<br><br>**Theories used:** No details<br><br>**Assessment of training:** No details<br><br>**Supervision:** No details	developed by the CHWs and reported that combining this model with self-sampling was convenient for them with their work and family responsibilities. For follow up of HPV positive women there were challenges: it occurred at 6 months however due to policy changes transportation of colposcopy equipment to Manchay was not possible; therefore, the 6-month follow-up for HPV positive patients was conducted by physicians at INEN rather than at the health clinic in Manchay. Each CHW travelled with the participants to INEN and assisted in facilitation of the follow-up visits. Several participants required two follow-up visits because, with the change of location, biopsies were not initially collected. Out of 29 women initially treated, 26 (90%) were seen and evaluated for the 6 month follow-up.	
Mandig	Pairing	2015	Haiti,	**Name:**	**Purpose:** To evaluate	**Content:** CHWs	CHWs approached 575	No details

o et al.	community health workers with HPV self-sampling for cervical cancer prevention in rural Haiti		Central America	Community Health Workers (CHWs)<br><br>**Number:** 13<br><br>**Description:** The CHWs were employed by PIH/ZL. CHWs are elected by their villages to discuss hygiene, nutrition, and women's health during monthly home visits. They had to be trusted and respected members of the community.<br><br>**Remuneration:** No details	the efficacy and acceptability of a program in which CHWs provided education and HPV self-sampling devices to Haitian women.<br><br>**Duration:** 24 months<br><br>**Role of CHW:** CHWs sensitised the women involved in the study and then demonstrated how to use the self-sampling. Results from the sampling were sent to the CHW supervisor and then disseminated to CHWs, who visited participants to give them results and instructions for follow-up in Cange or Thomonde.<br><br>**Screening modality:** HPV-DNA self-sampling	were trained about cervical cancer and HPV self-sampling using a manual developed previously with Haitian collaborators. The CHWs recommended using a flipchart during home visits, which was created using a community-based participatory research approach and tested by six focus groups of Haitian women.<br><br>**Duration:** 1 week<br><br>**Person or group responsible for delivering training:** No details	potentially eligible women and enrolled 493 (85.7%) Among the 485 women for whom questionnaires were received, 468 (96.5%) were comfortable using the self-sampler and 484 (99.8%) stated they would recommend it to others. Among 426 analysed samples, 54 (12.7%) were positive for high-risk HPV, of whom 46 (85.2%) received follow-up care and 17 (31.5%) had precursor lesions and were treated. This was within 3 years of testing positive for high-risk HPV.	

						**Theories used:** No details<br><br>**Assessment of training:** No details<br><br>**Supervision:** No details		
Mezei et al.	Community-based HPV self-collection versus visual inspection with acetic acid in Uganda: a cost-effectiveness analysis of the ASPIRE trial	2018	Uganda, Africa	**Name:** Community Health Workers (CHWs)<br><br>**Number:** No details<br><br>**Description:** No cadre description<br><br>**Remuneration:** No details	**Purpose:** To evaluate the cost effectiveness of (1) community-based (ie, home or workplace) self-collected HPV testing (facilitated by community health workers) with clinic-based visual inspection with acetic acid (VIA) triage of HPV-positive women ('HPV-VIA') and (2) clinic-based VIA ('VIA').<br><br>**Duration:** 5 years<br><br>**Role of CHW:** The role of CHWs in this study was to recruit community members and teach them about	**Content:** Training focussed on the trial protocol<br><br>**Duration:** No details<br><br>**Person or group responsible for delivering training:** No details<br><br>**Theories used:** No details<br><br>**Assessment of training:** No details	HPV-ST was the most effective and cost-effective screening strategy, reducing the lifetime absolute risk of cervical cancer from 4.2% (range: 3.8%–4.7%) to 3.5% (range: 3.2%–4%), 2.8% (range: 2.4%–3.1%) and 2.4% (range: 2.1%–2.7%) with ICERs of US$130 (US$110–US$150) per YLS, US$240 (US$210–US$280) per YLS, and US$470 (US$410–US$550) per YLS when performed one, three and five times per lifetime, respectively. Findings were robust across sensitivity analyses, unless HPV costs were more than quadrupled.	Yes. Formal cost-analysis was conducted. Monte Carlo simulation model of HPV infection and cervical cancer that was calibrated to epidemiological data from Uganda to project

					self-collection. CHWs would then transport the samples to local laboratories in Kampala, Uganda where they were tested for HPV.<br><br>**Screening modality:** HPV-DNA or VIA	**Supervision:** No details		the lifetime health and economic outcomes associated with self-collected HPV testing versus clinic-based VIA
Mittal et al.	HPV detection-based cervical cancer screening program in low-resource setting: lessons learnt from a community-based demonstration project in India	2015	India, South Asia	**Name:** Community Health Workers (CHWs)<br><br>**Number:** 4<br><br>**Description:** Female multi-purpose workers of the national reproductive health program were engaged as CHWs. They	**Purpose:** A demonstration project to assess feasibility of implementing HPV detection-based cervical cancer screening in primary care settings in India and to generate local evidence on feasibility and effectiveness of HPV detection in primary screening.<br><br>**Duration:** 5 years<br><br>**Role of CHW:** They were responsible for community mobilization and	**Content:** CHWs were trained to collect samples for the HPV-DNA testing and to perform VIA using the training materials developed by International Agency for Research on Cancer (IARC), Lyon, France.<br><br>**Duration:** 7 days<br><br>**Person or**	44,110 women were screened and HPV positivity was 4.7 %. Compliance to recall of HC2-positive women for colposcopy was 78 %. Detection rate of CIN3? by HPV test was 3.9/1,000 women. Compliance of women to treatment was 80.1 %. However, compliance of HPV-positive women for follow-up at 1 year was poor (23.2 %). Concurrent use of VIA to screen the women did not have any advantage but increased number of unnecessary colposcopies and biopsies.	No details

				were selected from villages in the implementation area and worked part-time until their respective villages had been covered. They were paid an incentive for the work.<br><br>**Remuneration:** No details	recalling the screen-positive women.<br><br>**Screening modality:** HPV-DNA and VIA	**group responsible for delivering training:** No details<br><br>**Theories used:** No details<br><br>**Assessment of training:** Competency based assessments<br><br>**Supervision:** In-service mentoring was done by a clinician for at least 1 month after completion of training. Refresher training of the HWs was held at least once a year.		
Srisuwan et al.	Knowledge, Attitudes and Practices Regarding Cervical	2015	Thailand, South-East Asia	**Name:** Village Health Volunteers (VHVs)	**Purpose:** To assess the knowledge and attitudes of cervical cancer screening among VHVs.	**Content:** No details<br><br>**Duration:** No details	The VHVs had a high level of knowledge about the cervical cancer screening. VHVs had positive attitudes to the promotion of cervical cancer	No details

	Cancer Screening Among Village Health Volunteers			**Number:** 128<br><br>**Description:** The VHVs provide basic health information and services to villagers. The volunteers are normal layman who receives organized health information from public health personals.<br><br>**Remunerati on:** No details	**Duration:** 2 months<br><br>**Role of CHW:** To provide health information and to conduct PAP screening.<br><br>**Screening modality:** PAP smear	**Person or group responsible for delivering training:** No details<br><br>**Theories used:** No details<br><br>**Assessment of training:** No details<br><br>**Supervision:** No details	screening. This study revealed barriers to screening however. The main barriers to screening were embarrassment, poor attitude, conflict, beliefs and socio-cultural. In Chomphol sub district, 95% of the target population were Muslim women. They held themselves strictly per the socio-cultural rule. Thai Muslim women were worried that the village Pap smear conducting area was not providing total privacy. They were bothered by the fact that the PAP test was perform to their private body parts by VHV who lived in the same village. They did not want to have their private part seen by their neighbours. Married women were not allowed to receive a PAP test without their husbands permission. As a result another screening barrier was their husbands' information and attitude toward this screening. In addition even though the VHVs had sufficient knowledge and	

							good attitudes, this wasn't enough to convince local women (especially Muslim women) to get screening. Therefore in some of the districts assessed, screening rates were as low as 47.3%, which was well below the target of 80% set by the government. Mobile screening services and the combination of cultural awareness training for health care professionals with outreach workers who could help healthcare professionals to overcome language and cultural barriers were likely to improve cancer screening uptake.	
Tum et al.	Creating awareness and facilitating cervical and breast cancer screening uptake through the use of a Community Health	2012	South Africa, Africa	**Name:** Community Health Workers (CHWs)<br><br>**Number:** 5<br><br>**Description:** To be selected by local community	**Purpose:** To develop and pilot an intervention involving a CHW to create awareness of cervical and breast cancer and to facilitate screening uptake in a specific resource poor community in Tshwane<br><br>**Duration:** 4 months	**Content:** The content was largely focussed on how to perform the role of a CHW and basic information on cervical cancer through a mix of group discussions, brainstorming	The target population of this study were women aged 30 years (n = 452) and older. Over an evaluation period of 4 months the screening uptake was 3.8%. The majority of the sample (81.8%) responded positively when asked if the CHW taught them about these cancers.	No details

	Worker: a pilot interventio n study			members and meet the following criteria: older than 18 years; able to under- stand, speak, read and write simple English; born and residing in the specific community; and completed secondary school (12 years of schooling).<br><br>**Remunerati on:** No details	**Role of CHW:** One month before data collection commenced, five CHWs were sent into the community to inform them about the study and the services available. During this time, the CHW specifically trained for cancer prevention was tasked to teach the community, especially the women, on what cancer is and what cervical and breast cancer are, the signs of cervical cancer breast cancer, the risk factors, prevention and early detection and the treatment of these diseases. CHWs were tasked to assist the women with undressing and dressing, ensuring that the women were suitably covered, supporting the woman during the screening procedure, assisting the registered nurse	and class assignments were used to foster better understanding.<br><br>**Duration:** Teaching sessions took place on Mondays to Wednesdays from 0800h to 1600h over a period of 3 months.<br><br>**Person or group responsible for delivering training:** The research team and a nurse.<br><br>**Theories used:** No details.<br><br>**Assessment of training:** A weekly written assignment was given		

					when performing the procedure and cleaning the instruments used for screening.<br><br>**Screening modality:** PAP smear	**Supervision:** No details		
Vallikad et al.	Cervical Cancer: The Indian Perspective	2006	India, South Asia	**Name:** Anganwadi Workers (AW) and Female Junior Health Volunteers (FJHVs)<br><br>**Number:** No details<br><br>**Description:** No details<br><br>**Remuneration:** No details	**Purpose:** To examine the feasibility of training different primary health centre (PHC) personnel to raise the awareness about cervical cancer in women aged between 35 and 64 years old, and conduct screening.<br><br>**Duration:** 24 months<br><br>**Role of CHW:** The FJHVs were trained to raise awareness. Some were also trained to perform Visual Inspection (VI), and identify the cervix as normal, abnormal (cervical polyp, reproductive tract infections etc.) and	**Content:** Information on awareness raising regarding cervical cancer and how to perform VI and smears.<br><br>**Duration:** One day for those just raising awareness, and an additional two days for those performing VIs and smears.<br><br>**Person or group responsible for delivering training:** Training was	There were many complaints from AWs and FJHVs and considerable indifference. Sustaining motivation was difficult. There was a demand for incentives (which were not given during the study).<br><br>The coverage of conducting was extremely poor, varying from 2.6% to 18.7%. Of the women who did undertake testing, the compliance was good (ranging from 64% at the Health Centre III to 98% at HC I (in the village)).<br><br>The personnel of the government health infrastructure can be trained to perform visual inspection and take a smear.	No details

					suspicious of malignancy (exophytic/ ulcerative lesions etc.). They were also trained in the technique of taking a smear. The FJHV could perform the VI or VI and cytology at a place convenient to her and the women, even at the homes of compliant women. No target was set for their work. They were expected to do these activities during the course of their regular work.<br><br>AWs were trained to impart health awareness.<br><br>**Screening modality:** PAP smear and VIA	conducted at the Regional Cancer Centre<br><br>**Theories used:** No details<br><br>**Assessment of training:** Pre- and post-training assessment<br><br>**Supervision:** FJHVs were supervised by the Lady Medical Officers at the Health Centres**,** AWs were supervised by an AW supervisor. The study support team provided monthly supervision support.	Although woman are interested in undergoing screening, the responsibility of imparting health awareness cannot be the sole responsibility of the PHC personnel. Furthermore compliance decreases if a woman has to access the PHC for the test, indicating the need to make the test available at Village Health Centres.<br><br>Cervical cytology, if undertaken by the FJHV, is cumbersome: taking an adequate smear; fixing the smear; labelling the smear; preserving the slides; transporting the slides to the PHC; collection and transport to the laboratory; processing the smears; reporting the slides; quality control of the laboratory; transporting the reports to the PHC; distributing the reports to the individual women; compliance of the women with the results of cytology for treatment and follow-up.	

Legend: Key data extraction points for each of the studies included in the review.
